# Development of a Fluorescence Polarization Assay for Multi-Determination of 10 Aminoglycosides in Pork Muscle Sample Based on Ribosomal Protein S12 and Studying Its Recognition Mechanism

**DOI:** 10.3390/foods11203196

**Published:** 2022-10-13

**Authors:** Wanqiu Xia, Lei Zhang, Jianping Wang

**Affiliations:** 1 College of Veterinary Medicine, Hebei Agricultural University, Baoding 071000, China; 2 Veterinary Biological Technology Innovation Center of Hebei Province, Baoding 071000, China

**Keywords:** aminoglycosides, ribosomal protein S12, recognition mechanism, fluorescence polarization assay, pork muscle

## Abstract

The residues of aminoglycosides in foods of animal origin are a potential risk to consumers. There have been some immunoassays reported for the screening of aminoglycoside residues, but the method showing the broadest detection spectrum can only be used to detect two drugs. This is because a broad specific recognition reagent is not available. In the present study, the receptor of aminoglycosides (ribosomal protein S12 of *Lysinibacillus* *sphaericus*) was expressed, and its affinities and recognition mechanisms for 10 aminoglycosides were studied by using surface plasmon resonance and molecular docking, respectively. Then the receptor was used as a recognition reagent to develop a fluorescence polarization assay on a 96-well microplate for the detection of the 10 drugs in pork muscle samples. The limits of detection for the 10 drugs ranged from 5.25 to 30.25 ng/g. The sensitivities for the 10 drugs were generally consistent with their respective receptor affinities and binding energies. After comprehensive comparison, the method performances were better than all the previously reported immunoassays for aminoglycosides. This is the first study reporting the recognition mechanisms of ribosomal protein S12 of *Lysinibacillus* *sphaericus* for 10 aminoglycosides and the use of it as a recognition reagent to develop a pseudo-immunoassay for the multi-determination of aminoglycosides in food samples.

## 1. Introduction

**Figure 1:**
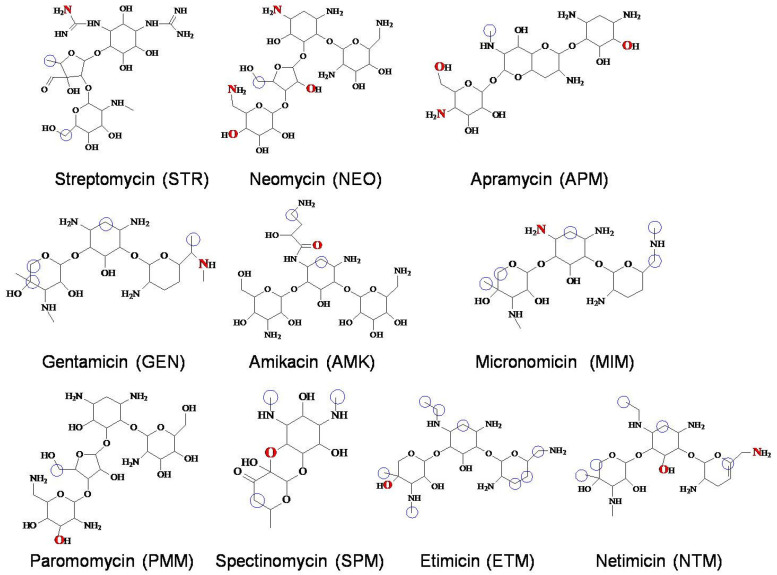
Molecules of the 10 AGs. (Atoms highlighted inred were the binding sites of hydrogen bond. Atoms highlighted with blue circles were the binding sites of hydrophobic interaction.).

Aminoglycoside drugs (AGs) have been used for the treatment of Gram-negative bacteria-induced infections in human beings and animals for nearly 80 years. The molecules of 10 commonly used AGs are shown in Figure 1. However, the extensive use of AGs in food-producing animals inevitably leads to their residues in foods of animal origin that are potential risks to the consumers due to their severe nephrotoxicity and ototoxicity [1,2]. Therefore, many methods have been reported to detect AG residues in different food samples; the instrumental method and immunoassay are the commonly used techniques [3,4].

In comparison with instrumental methods, immunoassays are simple and cheap, so they are usually used for the rapid screening of a large number of samples. By now, there have been different types of immunoassays reported to detect AGs, including enzyme-linked, immunosorbent assays [5,6,7,8,9,10,11], fluorometric immunoassays [12], chemiluminescent immunoassays [13], immunochromatographic strips [14,15,16,17], and biosensors [18].

It is well known that the key element of immunoassays is used as a recognition reagent. Among the reported immunoassays for AGs, polyclonal antibodies, monoclonal antibodies, and aptamers are commonly used recognition reagents. However, the recognition abilities of these reagents are limited. For example, most of the antibodies [6,8,9,12,13,14,15,16,17] and all the aptamers [18] can only recognize one drug, and the antibodies showing the broadest recognition spectrum can recognize at most two Ags [5,10,11]. This means that an immunoassay capable of multi-determining Ags residues has not been reported so far, which is because a broad specific recognition reagent for Ags is not available. Therefore, it is necessary to find new recognition reagents with broad recognition ability for Ags.

It is well known that the receptor is a natural macromolecule, and a specific receptor is the usual target site of one class of drugs, so in theory, it should recognize all the species of this class of drugs. During the past few years, some receptors have been used as recognition reagents to develop pseudo-immunoassays for the detection of sulfonamides [19,20,21,22,23], β-lactams [24], tetracyclines [25,26,27], and β_2_-agonists [28]. Results showed that these receptors exhibited broader detection spectra than the controlled antibodies. For example, our recently reported pseudo-immunoassay based on dihydropteroate synthase could determine 40 sulfonamides [23], better than all the antibody-based immunoassays.

The prokaryotic ribosome is composed of the 50S and 30S subunits, and the latter is composed of 16S rRNA and 21 ribosomal proteins. AGs can bind 16S rRNA helices and ribosomal protein S12 (*RpsL*12) to interfere with the function of the bacterial ribosome and show an antibacterial effect [29,30,31]. Furthermore, the amino acid mutations in *RpsL*12 have been proven to be the major mechanism of AG-resistant bacterial strains [32,33,34,35]. Therefore, *RpsL*12 is the AG receptor that should recognize and bind to all AG species. To the best of our knowledge, however, there has been no study reporting the use of *RpsL*12 as a recognition reagent for the detection of AGs so far. In addition, all the previously reported studies on *RpsL*12 focus on studying the AG antibacterial and AG resistant mechanisms, and interaction mechanisms with AGs have not been studied.

Among the reported different immunoassays, the fluorescence polarization immunoassay is very simple and contains only one sample-loading step, so one assay can be finished in several minutes. As a result, this method has been used for the determination of many analytes [36]. In the present study, the *Lysinibacillus* *sphaericus* *RpsL*12 was expressed, and its recognition mechanisms and affinities for the 10 AGs, shown in Figure 1, were studied by using the molecular docking and surface plasmon resonance, respectively. Then it was used as a recognition reagent to develop a fluorescence polarization assay (FPA) for the multi-determination of 10 AGs in a pork muscle sample.

## 2. Materials and Methods

### 2.1. Reagents and Chemicals

The standards of streptomycin (STR), neomycin (NEO), gentamicin (GEN), amikacin (AMK), spectinomycin (SPM), apramycin (APM), paromomycin (PMM), netimicin (NTM), and fluorescein isothiocyanate (FITC) were from Shanghai Yuanye Biological Technology Co., Ltd. (Shanghai, China). Micronomicin (MIM) and etimicin (ETM) were from the China National Institute for Food and Drug Control (Beijing, China). All the used biological reagents were the same as those in our recent report [25] (see Supplementary Materials).

### 2.2. Expression of RpsL12

The recombinant plasmids were synthesized at Sangon Biotech Co., Ltd. (Shanghai, China) by inserting the *RpsL*12 gene of *Lysinibacillus* *sphaericus* (GenBank ID: AHC30887.1) into the express vector pET32a(+). The plasmids containing the express vector pET32a-*RpsL*12 were transformed into BL21 (D3) for acculturation, and the gene products in the positive colonies were characterized by a polymerase chain reaction and DNA sequencing. Then the *RpsL*12 was expressed with the same procedures as those described in our recent report [25] (see Supplementary Materials), and the obtained product was analyzed with SDS-PAGE electrophoresis and Western blotting.

### 2.3. Molecular Docking

The amino acid sequence of *L. sphaericus RpsL*12 was translated from its gene sequence by using DNAman 6.0 (LynnonBiosoft, San Ramon, CA USA) and compared with the amino acid sequences of several *RpsL*12 from other bacterial strains. Then its 100% homological 3D conformation (*E.* *coli* *RpsL*12, PDB ID:7P7S) was used to dock with the 10 AGs, and their intermolecular interaction mechanisms were determined, including the binding pocket, binding energy, contact amino acid, intermolecular force, and binding site.

### 2.4. Surface Plasmon Resonance (SPR)

The affinities of the *RpsL*12 for the 10 AGs were determined by using the surface plasmon resonance technique (SPR), and the assay procedures were performed according to our recent report [22] (see Supplementary Materials). During the SPR test, the association constant (Ka), dissociation constant (Kd), and equilibrium dissociation constant (KD = Kd/Ka) for the 10 AGs were determined. Furthermore, the absolute affinity constants (KA=Log2(KD)) for the 10 AGs were also calculated, which were used to conveniently compare their affinities.

### 2.5. Synthesis of Fluorescent Tracer STR-FITC

**Figure 2:**
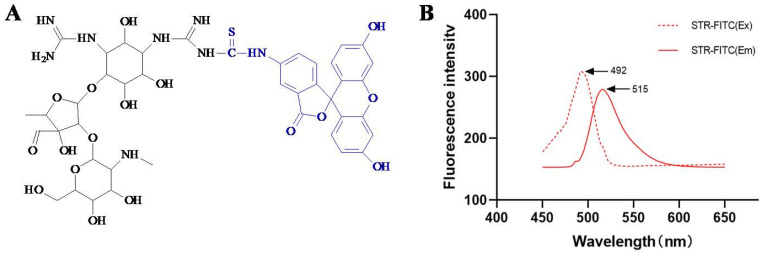
(**A**) Molecule of STR-FITC and (**B**) its fluorescence spectrum. STR-FITC is the conjugate of streptomycin (STR) and fluorescein isothiocyanate (FITC). STR-FITC (Ex) is the excitation wavelength of STR-FITC, and STR-FITC (Em) is emission wavelength of STR-FITC.

The fluorescent tracer was synthesized by coupling streptomycin (STR) with fluorescein isothiocyanate (FITC) (Figure 2). Briefly, 12 mg (20 μmol) STR and 25 μL triethylamine were added into 1 mL water. Eight mg (20 μmol) FITC, dissolved in 200 μL DMSO, was added into the above solution to be gently stirred at room temperature for 12 h. Then the mixture was transferred onto a homemade thin-layer chromatography plate and separated by using methanol: benzene (1:3, *v*/*v*). The silica gel band containing the target (Rf value of 0.14) was scraped, and the obtained powder was washed with 3 mL methanol. The yellow-green eluate containing STR-FITC (4 mg/mL) was collected and stored at −4 °C for use.

### 2.6. Development of Fluorescence Polarization Assay (FPA)

The analyte solution (50 μL), the fluorescent tracer solution (50 μL), and the *RpsL*12 solution (50 μL) diluted with PBS (pH 7.0) were added to the wells of a 96-well microplate. The plate was incubated for 3 min, and the fluorescence polarization value (*FP*) of each well was recorded at λ_ex485_/λ_em528_ nm (emission cutoff of 515 nm). During the experiments, the *RpsL*12 concentration, tracer concentration, incubation time, and pH of the assay solution were optimized. Then the competitive inhibition curves for the 10 AGs were developed by plotting the *FP/FP_0_* values (where *FP* was the fluorescence polarization value of each concentration and *FP_0_* was the fluorescence polarization value of zero concentration) versus the drug concentrations (Log C). Half of the inhibition concentration (IC_50_, the analyte concentration showing 50% inhibition) and limit of detection (IC_10_, the analyte concentration showing 10% inhibition) for each drug were calculated.

### 2.7. Sample Extraction and Analysis

The AG residues in the pork muscle sample were extracted as follows. Before sample preparation, the fat tissues in the muscle samples were removed as fully as possible. Then 2 g of homogenized pork samples and 5 mL of 3% trichloroacetic acid were added into a centrifuge tube to be stirred for 5 min. After centrifugation at 10,000 rpm for 5 min, the supernatant was neutralized with 30% NaOH solution. Finally, an appropriate volume of the solution was transferred onto the microplate for analysis. During the experiments, some blank pork muscle samples, which were known to be free from AGs, were collected from several controlled slaughterhouses to evaluate the method. The 10 AGs were fortified into the blank samples at different levels to be extracted and analyzed. Finally, 50 real pork muscle samples were collected from some local supermarkets and analyzed as described above.

## 3. Results and Discussions

### 3.1. Characterization of RpsL12

**Figure 3:**
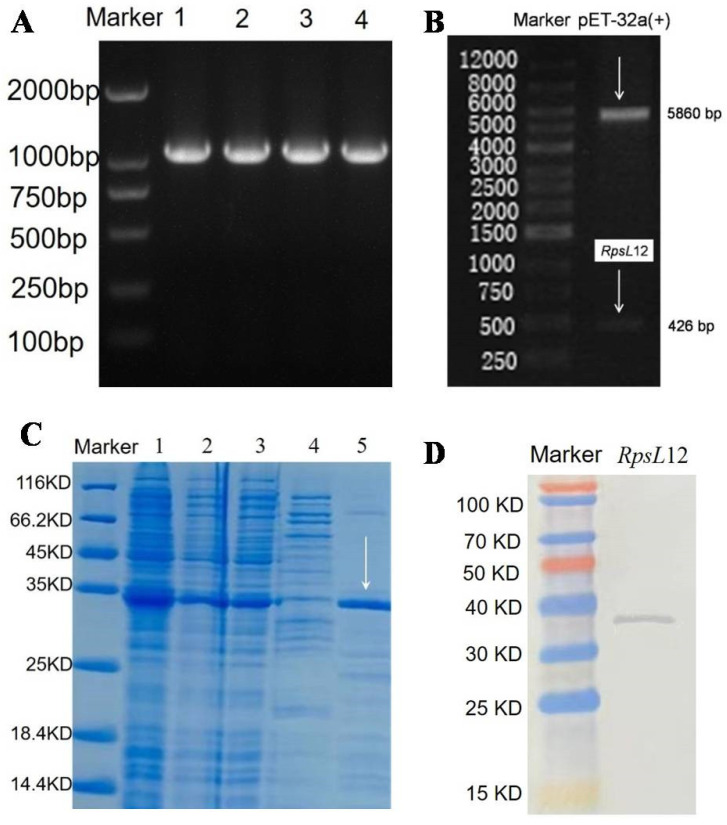
(**A**) Agarose gel electrophoresis results of (**A**) *RpsL*12 gene (1138 bp, containing *RpsL*12 gene 426 bp and T7 primer 712 bp) (lane 1-4, PCR products) and (**B**) express vector pET32a-*RpsL*12 after enzyme digestion. (**C**) SDS-PAGE results of *RpsL*12 (34 kDa) (lane 1, bacterial whole protein; lane 2, supernatant; lane 3, inclusion body; lane 4, collected rinse solution during supernatant purification; lane 5, purified supernatant). (**D**) Western blotting analysis of *RpsL*12.

In this study, the *L. sphaericus RpsL*12 gene was directly inserted into pET32a(+) to synthesize the expression vector. As shown in Figure 3A, the expected *RpsL*12 gene was obtained (1138 bp, containing the target *RpsL*12 gene 426 bp and the T7 primer 712 bp). As shown in Figure 3B, the expected genes of pET32a(+) (5860 bp) and *RpsL*12 (426 bp) after enzyme digestion were also obtained. Then the pET32a-*RpsL*12 was expressed in *Escherichia coli* BL21(D3). As shown in Figure 3C, the *RpsL*12 was expressed in both the inclusion body and the supernatant (molecular weight 34 kDa). For convenience, the *RpsL*12 in the supernatant was purified for the subsequent experiments. As shown in Figure 3D, the results of a Western blotting experiment showed the target protein was obtained. Furthermore, its 100% conserved amino acids were the same as the *RpsL*12 from other bacterial strains (Figure S1, Supplementary Materials). These results showed that the target *RpsL*12 was obtained.

### 3.2. Recognition Mechanisms for AGs

**Figure 4:**
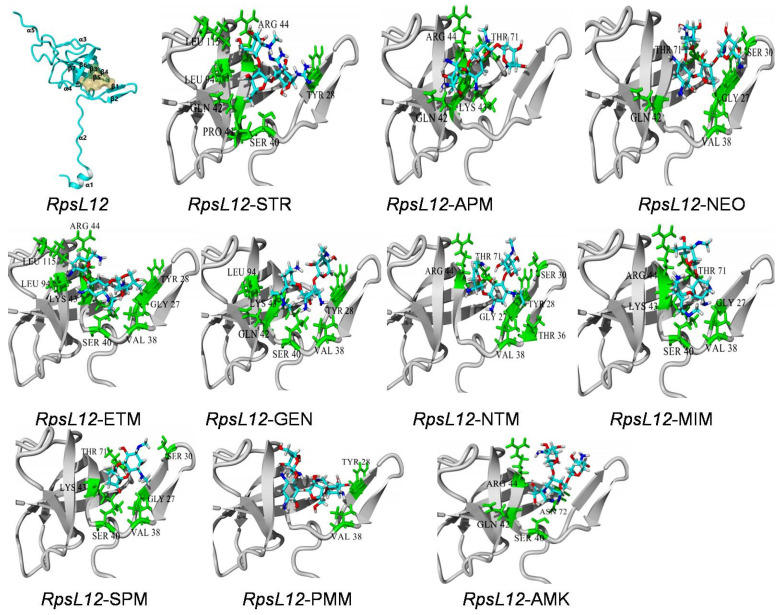
General conformation of *RpsL*12 and the close-up view of its docking complexes with the 10 AGs.

As far as we know, there has been only one study reporting the intermolecular interaction of *L. sphaericus RpsL*12 with AGs (streptomycin) [35]. The results showed that the amino acids at the 40–45 positions constructed the binding pocket, and hydrogen bonds (Thr40, Pro41, Arg42, Lys43, Asn45) and hydrophobic interaction (Pro41 and Pro44) were the main intermolecular forces. However, other intermolecular interaction parameters and their interaction mechanisms with other AGs were not studied. In the present study, the 100% homological model of the present *L. sphaericus RpsL*12 (PDB ID: 7P7S) was docked with the 10 AGs to study its recognition mechanisms, and the docking complexes and docking results were shown in Table 1 and Figure 1 and Figure 4.

As shown in Figure 4, the present *RpsL*12 contained five α-helixes and seven β-sheets, and the binding pocket was surrounded by β1, β2, β3, and β4. The 10 AGs could all be docked into the pocket. STR, GEN, AMK, MIM, ETM, SPM, NTM and NEO were deep in the pocket, whereas PMM and APM only interacted with one side wall of the pocket. The molecules of STR, GEN, AMK, MIM, ETM, PMM, NTM, and NEO were generally in a “V shape” in the pocket, whereas the molecules of APM and SPM were in plane formations. As shown in Table 1, the hydrophobic interaction was the main intermolecular force, and the hydrogen bond was the secondary force, though the specific contact amino acids for the 10 drugs were not the same.

General considerations of the results are shown in Table 1 and Figure 4. VAL38 from β2 (interacting with 7 AGs) and SER40 from β3 (interacting with 6 AGs) constructed the pocket bottom; GYL27 from β1 (interacting with 5 AGs) and TYR28 from β1 (interacting with 6 AGs) constructed one side of the pocket, and GLN42 from β3 (interacting with 5 AGs), LYS43 from β3 (interacting with 5 AGs) and ARG44 from β3 (interacting with 6 AGs) and THR71 from β4 (interacting with 5 AGs) constructed the other side of the pocket. The positions of these contact amino acids were similar to the previous report [35]. As shown in Figure 1, the specific binding sites in the 10 AGs were different, and this may be because of their different molecular structures. Still, the binding energies of the *RpsL*12 for the 10 drugs were in a narrow range (5.15–5.84 kcal/moL, Table 1), indicating that the *RpsL*12 showed comparable recognition for them.

For confirmation of the molecular docking results, the receptor affinities for the 10 drugs were also determined by SPR. The detailed Ka, Kd, KD, and KA are shown in Table S1 (Supplementary Materials), and the KA values are also shown in Table 1. As shown in Table 1, the KA values for the 10 drugs ranged from 11.358–26.102, which meant the present *RpsL*12 showed comparable affinities to the 10 drugs, consistent with the docking results. The recognition mechanisms of *RpsL*12 protein for AGs reported in the present study were more comprehensive than the previous report [35].

### 3.3. Characterization of the Fluorescent Tracer

For the development of a fluorescence polarization assay, a fluorescent tracer was required. In the present study, STR was coupled with FITC to synthesize the fluorescent tracer STR-FITC (Figure 2A). As shown in Figure 2B, its excitation wavelength was 492 nm, and its emission wavelength was 515 nm, indicating that the STR-FITC was synthesized.

### 3.4. Evaluation of RpsL12 and STR-FITC

**Figure 5:**
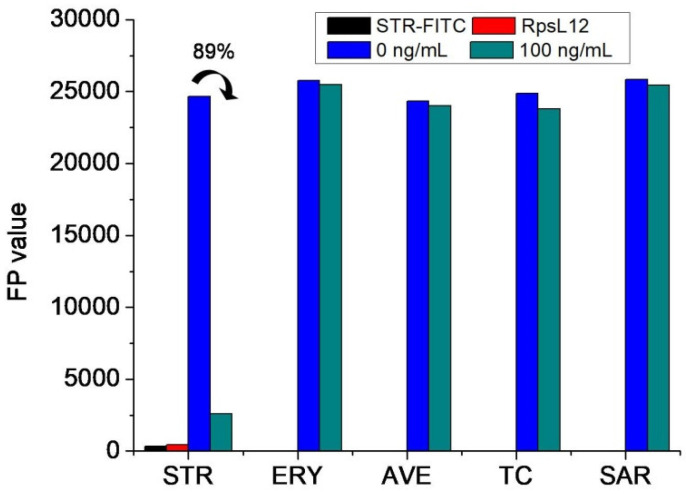
*FP* values when testing STR and other drugs by using STR-FITC (8 μg/mL) and *RpsL*12 (2 μg/mL) (pH 7.0; incubation time 5 min; ERY = erythromycin, AVE = avermectin, TC = tetracycline, SAR = sarafloxacin).

For evaluating if the above two reagents could be used to develop an FPA method, the *RpsL*12, the STR-FITC, and five drugs (STR, erythromycin, avermectin, tetracycline, and sarafloxacin; 0 and 100 ng/mL) were mixed to perform the assay. As shown in Figure 5, when the concentration of these drugs was at 0 ng/mL, the *FP* values of single *RpsL*12 and single STR-FITC were all negligible, but the *FP* values of *RpsL*12+STR-FITC were high. This proved that the STR-FITC could bind with the *RpsL*12 to induce the fluorescence polarization phenomenon, i.e., they could be used to develop an FPA method. As shown in Figure 5, when the concentration of these drugs was at 100 ng/mL, the *FP* value, when detecting STR, largely decreased (inhibition ratio 89%), but the detection of other drugs showed a liter change (inhibition ratios < 4%). These results proved that the *RpsL*12 was only specific for STR. So, the two reagents were used to optimize the FPA method.

### 3.5. Optimization of FPA Method

**Figure 6:**
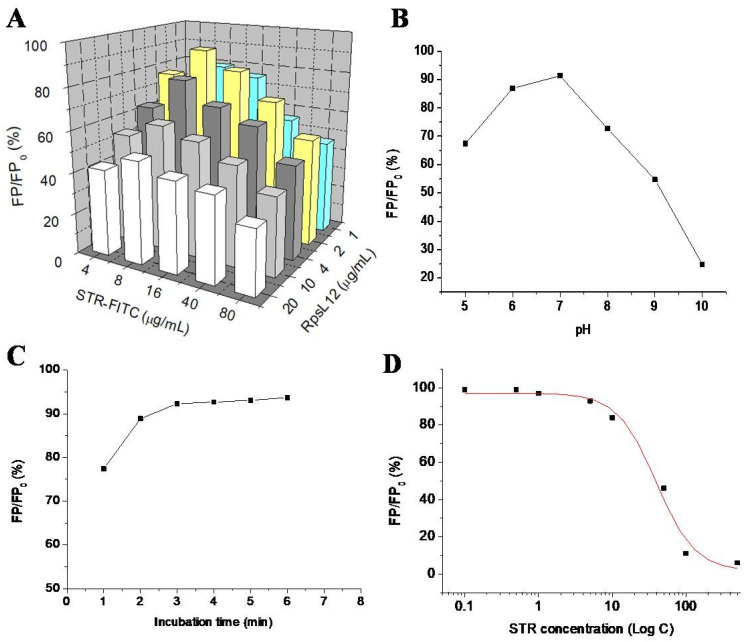
Results for optimization of (**A**) *RpsL*12/STR-FITC concentration (pH 7.0, incubation 5 min), (**B**) pH (STR-FITC 8 μg/mL, *RpsL*12 2 μg/mL, incubation 5 min), and (**C**) incubation time (STR-FITC 8 μg/mL, *RpsL*12 2 μg/mL, pH 7.0) by using STR (100 ng/mL). (**D**) Competitive inhibitory curve of STR (0.1–500 ng/mL).

This is the first study reporting the use of *RpsL*12 as a recognition reagent to develop an FPA method for the detection of AGs. Several parameters were optimized with STR as the representative to obtain the best-performing method. Firstly, the concentrations of *RpsL*12 and STR-FITC were optimized. During the experiments, different concentrations of *RpsL*12 and STR-FITC were mixed with an STR solution (100 ng/mL) to perform the assay. As shown in Figure 6A, the inhibition ratio of STR was the highest when the concentrations of *RpsL*12 and STR-FITC were 2 and 8 μg/mL, respectively. Secondly, the pH of the assay system was optimized. During the experiments, STR and *RpsL*12 were diluted with PBS at different pH values to perform the assay. As shown in Figure 6B, the inhibition ratio of STR was the highest when the assay system was at a pH of 7.0. Thirdly, the competition time was optimized. As shown in Figure 6C, the inhibition ratio of STR reached a plateau when the mixture was incubated for 3 min. Therefore, the above optimal parameters were used for the following experiments.

### 3.6. Method Performances

Under optimal conditions, the 10 AGs were diluted with the extracts of blank pork muscle samples to be assayed by the FPA method. The representative competitive inhibition curve of STR is shown in Figure 6D. As shown in Table 1, the IC_50_ values for the 10 AGs were in the range of 47.6–72.2 ng/mL, and the limits of detection (IC_10_) were in the range of 2.1–12.1 ng/mL. Due to the 2.5-fold dilution during sample preparation, the limits of detection for the determination of the 10 AGs in the pork muscle sample were in the range of 5.25–30.25 ng/g.

**Figure 7:**
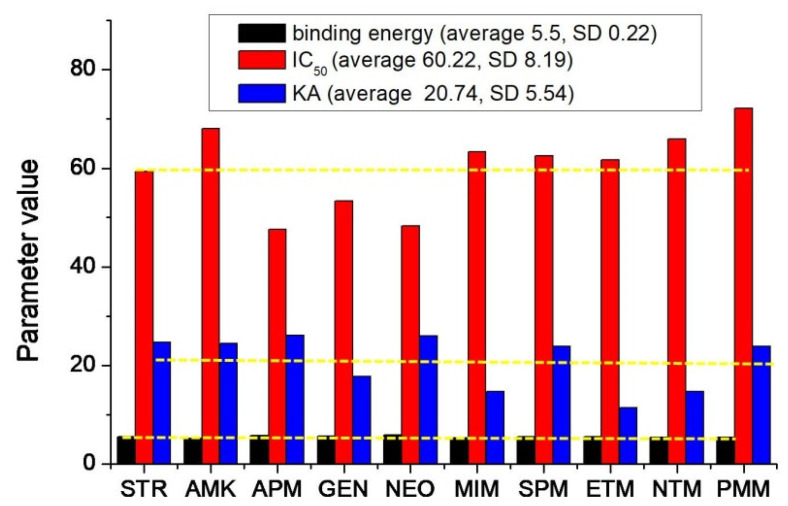
Change trends of the binding energy, absolute affinity constants (KA), and IC50 for the 10 AGs. (The yellow line is the average value of each parameter).

For an overview of the method sensitivity and *RpsL*12 recognition ability, the binding energy (5.15–5.84 kcal/moL), KA (11.358–26.102), and IC_50_ (47.6–72.2 ng/mL) of the 10 AGs were integrated. As shown in Figure 7, the average value of the binding energy was 5.5 with a standard deviation of 0.22, the average value of KA was 60.22 with a standard deviation of 8.19, and the average value of IC_50_ was 20.74 with a standard deviation of 5.54. This meant that the three parameters for the 10 drugs were all in a narrow range, i.e., the *RpsL*12 showed comparable bindings, affinities, and sensitivities to the 10 drugs, which was the base to develop the multi-analytes FPA method for the 10 AGs.

### 3.7. Sample Determination

For the evaluation of the method of application, the 10 AGs were fortified in the blank pork muscle samples to be assayed. As shown in Table 2, the recoveries were in the range of 74.5–97.6% and the coefficients of variation were in the range of 6–14%. Finally, the 50 real pork muscle samples were analyzed by the FPA. It was found that only one real sample was determined as positive by the present method, and the residue level was expressed as STR with 26 ± 4.1 ng/g (3 repetitions), which was lower than its maximum residue limit in meat, 100 ng/g [37]. However, the specific drug species could not be identified because of the *RpsL*12’s broad recognition ability. Therefore, the positive results of the present FPA method for real samples should be confirmed by using an instrumental method (e.g., LC-MS/MS) to identify the specific AGs species, but such an instrumental method capable of simultaneous determining the 10 AGs remained to be studied. Nevertheless, the present FPA method could be used as a simple screening tool for the multi-detection of the 10 AGs in many pork muscle samples.

### 3.8. Comparison with Related Immunoassays

This study used *RpsL*12 as a recognition reagent to develop an FPA method for the multi-determination of 10 AGs. For comparison, some previously reported immunoassays for the detection of AGs [5,6,7,8,9,10,11,12,13,14] are listed in Table 3. First, the detection spectrum of the present FPA method was broader than all those methods. Second, the operation process of the present FPA was simpler than all those methods. Third, the assay time of the present FPA method was shorter than all those methods. Fourth, the sensitivity of the present FPA method was not the highest, but it could still be used to detect low levels of AG residues. With a general consideration of these points, the present FPA method showed a better performance than those methods.

## 4. Conclusions

The residues of AGs in foods of animal origin are potential risks to consumers, and many immunoassays have been reported to detect their residues. All the previously reported immunoassays cannot multi-determine this class of drugs, and the immunoassay showing the broadest detection spectrum can only determine two drugs. This present study expressed *RpsL*12 and used it as a recognition reagent to develop an FPA method for the multi-determination of 10 AGs in pork muscle samples. Results showed that the method performances were generally better than all the previous immunoassays for AGs. Therefore, this method could be used as a simple, rapid, and sensitive tool for multi-screening the residues of the 10 AGs in a large number of food samples.

## Figures and Tables

**Table 1:** Docking results, absolute affinity constants (KA) and determination parameters for the 10 AGs.

Drug	Binding Energy (kcal/mol)	Contact Amino Acid		KA	IC_50_ (ng/mL)	LOD (ng/mL)
		Hydrogen Bond	Hydrophobic Interaction			
STR	5.56	TYR28	SER40 PRO41 GLN42 AGR44 LEU94LEU115	24.670	59.3	7.2
AMK	5.15	ARG44	SER40 GLN42 ASN72	24.487	68.1	11.3
APM	5.78	GLN42 THR71	LYS43 ARG44 THR71	26.102	47.6	3.6
GEN	5.64	GLN42 LYS43	TYR28 VAL38 SER40 GLN42 LEU94	17.693	53.3	2.1
NEO	5.84	TYR28 SER30 GLN42 THR71	GLY27 VAL38	25.997	48.3	3.4
MIM	5.26	LYS43	GLY27 VAL38 SER40 ARG44 THR71	14.666	63.3	9.8
SPM	5.55	THR71	GLY27 SER30 VAL38 SER40 LYS43	23.900	62.5	9.2
ETM	5.57	TYR28 SER40	GLY27 VAL38 SER40 LYS43 ARG44 LEU94 LEU115	11.358	61.7	6.5
NTM	5.38	THR71	GLY27 TYR28 SER30 THR36 VAL38 ARG44	14.684	65.9	7.0
PMM	5.36	TYR28	VAL38	23.851	72.2	12.1

**Table 2:** AGs recoveries from the standards fortified blank pork muscle samples (*n* = 5).

Drug	Added (ng/g)	Inter-Assay		Intra-Assay	
		Recovery (%)	CV (%)	Recovery (%)	CV (%)
streptomycin	10	78.6	9	79.5	6
	100	86.3	9	75.3	6
neomycin	10	86.5	9	82.5	8
	100	92.5	11	84.3	7
gentamicin	10	92.4	13	92.1	7
	100	95.1	8	94.5	7
amikacin	20	76.5	9	91.2	7
	100	80.4	14	97.6	7
spectinomycin	10	91.5	10	89.3	8
	100	93.1	9	92.2	8
apramycin	10	85.8	8	96.5	7
	100	84.3	11	94.6	6
paromomycin	20	76.8	8	88.5	7
	100	84.7	8	83.2	8
netimicin	10	79.6	9	91.2	6
	100	82.6	9	92.8	8
micronomicin	10	82.5	12	90.7	6
	100	74.5	8	92.6	6
etimicin	10	80.6	8	86.4	8
	100	82.8	13	88.5	7

**Table 3:** Comparisons with some previous immunoassays for detection of AGs.

Recognition Reagent	Method	Analyte	Assay Time (from Add Sample)	LOD (ng/g)	Ref.
Kanamycin mAb	ELISA	2 AGs	30 min	0.9–1.8	5
Streptomycin pAb	ELISA	1 drug	30 min	1	6
AGs derivative pAb	ELISA	3 derivatives	>14 h	1–20	7
Gentamycin pAb	ELISA	1 drug	2 h	14.16	8
Apramycin mAb	Immunoaffinity test column	1 drug	20 min	3–10	9
Kanamycin mAb	ELISA	2 AGs	100 min	0.022	10
Streptomycin mAb	ELISA	2 AGs	40 min	0.09–1.37	11
Streptomycin mAb	Fluorescence ELISA	1 drug	60 min	0.005	12
Commercial Ab	Chemiluminescent ELISA	1 drug	60 min	9.4	13
Neomycin mAb	ELISA	1 drug	80 min	2.73	14
*RpsL*12	FPA	10 AGs	3 min	5.25–30.25	This study

## Data Availability

The data presented in this study are available on request from the corresponding author.

## Supplementary Materials

The following supporting information can be downloaded at: https://www.mdpi.com/article/10.3390/foods11203196/s1. Figure S1: Amino acid sequences of the present *RpsL*12 (Lysinibacillus sphaericus, PDB: 7P7S) and the *RpsL*12 proteins of Bacillus subtili (PDB: 3J9W), Escherichia coli (PDB: 5JTE), Enterococcus faecalis (PDB: 7P7U), Thermus thermophilus HB8 (PDB: 2F4V), and Acinetobacter baumannii (PDB: 7RYF). The amino acids highlighted in dark blue are the 100% conserved residues; Table S1: Ka, Kd, KD and KA values of the *RpsL*12 for the 10 AGs.

## References

[1] Aronson J.K., Reynolds D.J. (1992). ABC of monitoring drug therapy, aminoglycoside antibiotics. BMJ.

[2] Selimoglu E. (2007). Aminoglycoside-induced ototoxicity. Curr. Pharm. Des..

[3] Farouk F., Azzazy H.M.E., Niessen W.M.A. (2015). Challenges in the determination of aminoglycoside antibiotics, a review. Anal. Chim. Acta.

[4] Hari R., Taherunnisa S., Raut S.Y., Mutalik S., Koteshwara K.B. (2019). Challenges in the development of analytical test procedure for aminoglycosides: A critical review. J. Appl. Pharm. Sci..

[5] Chen Y., Wang Z., Wang Z., Tang S., Zhu Y., Xiao X. (2008). Rapid enzyme-linked immunosorbent assay and colloidal gold immunoassay for kanamycin and tobramycin in swine tissues. J. Agric. Food Chem..

[6] Abuknesha R.A., Luk C. (2005). Enzyme immunoassays for the analysis of streptomycin in milk, serum and water: Development and assessment of a polyclonal antiserum and assay procedures using novel streptomycin derivatives. Analyst.

[7] Shalev M., Kandasamy J., Skalka N., Belakhov V., Rosin-Arbesfeld R., Baasov T. (2013). Development of generic immunoassay for the detection of a series of aminoglycosides with 6′-OH group for the treatment of genetic diseases in biological samples. J. Pharmaceut. Biomed..

[8] Ho T.Y.J., Chan C., Chan K., Wang Y.C., Lin J., Chang C., Chen C. (2013). Development of a novel bead-based 96-well filtration plate competitive immunoassay for the detection of Gentamycin. Biosens. Bioelectron..

[9] Xu F., Jiang W., Zhou J., Wen K., Wang Z., Jiang H., Ding S. (2014). Production of monoclonal antibody and development of a new immunoassay for apramycin in food. J. Agric. Food Chem..

[10] Jiang L., Wei D., Zeng K., Shao J., Zhu F., Du D. (2018). An enhanced direct competitive immunoassay for the detection of kanamycin and tobramycin in milk using multienzyme-particle amplification. Food Anal. Methods.

[11] Wei D., Meng H., Zeng K., Huang Z. (2019). Visual dual dot immunoassay for the simultaneous detection of kanamycin and streptomycin in milk. Anal. Methods.

[12] Song E., Yu M., Wang Y., Hu W., Cheng D., Swihart M.T., Song Y. (2015). Multi-color quantumdot-based fluorescence immunoassay array for simultaneous visual detection of multiple antibiotic residues in milk. Biosens. Bioelectron..

[13] Luo P.J., Zhang J.B., Wang H.L., Chen X., Wu N., Zhao Y.F., Wang X.M., Zhang H., Zhang J.Y., Zhu L. (2016). Rapid and sensitive chemiluminescent enzyme immunoassay for the determination of neomycin residues in milk. Biomed. Environ. Sci..

[14] Jin Y., Jang J., Lee M., Han C. (2006). Development of ELISA and immunochromatographic assay for the detection of neomycin. Clin. Chim. Acta.

[15] Hendrickson O.D., Byzova N.A., Zvereva E.A., Zherdev A.V., Dzantiev B.B. (2021). Sensitive lateral flow immunoassay of an antibiotic neomycin. J. Food Sci. Technol..

[16] Sun Y., Yang J., Yang S., Sang Q., Teng M., Li Q., Deng R., Feng L., Hu X., Zhang G. (2018). Development of an immunochromatographic lateral flow strip for the simultaneous detection of aminoglycoside residues in milk. RSC Adv..

[17] Wei D., Zhang X., Chen B., Zeng K. (2020). Using bimetallic Au@Pt nanozymes as a visual tag and as an enzyme mimic in enhanced sensitive lateral-flow immunoassays: Application for the detection of streptomycin. Anal. Chim. Acta.

[18] Yue F., Li F., Kong Q., Guo Y., Sun X. (2021). Recent advances in aptamer-based sensors for aminoglycoside antibiotics detection and their applications. Sci. Total Environ..

[19] Liang X., Wang Z., Wang C., Wen K., Mi T., Zhang J., Zhang S. (2013). A proof-of-concept receptor-based assay for sulfonamides. Anal. Biochem..

[20] Wang Z., Liang X., Wen K., Zhang S., Li C., Shen J. (2015). A highly sensitive and class-specific fluorescence polarisation assay for sulphonamides based on dihydropteroate synthase. Biosens. Bioelectron..

[21] Liang X., Li C., Zhu J., Song X., Yu W., Zhang J., Zhang S., Shen J., Wang Z. (2019). Dihydropteroate synthase based sensor for screening multi-sulfonamides residue and its comparison with broad-specific antibody based immunoassay by molecular modeling analysis. Anal. Chim. Acta.

[22] He T., Liu J., Wang J.P. (2021). Development of a dihydropteroate synthase based fluorescence polarization assay for detection of sulfonamides and studying its recognition mechanism. J. Agric. Food Chem..

[23] He T., Cui P.L., Liu J., Feng C., Wang J.P. (2022). Production of a natural dihydropteroate synthase and development of a signal amplified pseudo immunoassay for determination of sulfonamides in pork. J. Agric. Food Chem..

[24] Ahmed S., Ning J., Cheng G., Maan M.K., Chen T., Ahmad I., Algharib S.A., Yuan Z. (2020). Development and validation of an enzyme-linked receptor assay based on mutant protein I188K/S19C/G24C for 40 beta-lactams antibiotics detection in 13 food samples. Microchem. J..

[25] Wang G., Zhang H.C., Liu J., Wang J.P. (2019). A receptor-based chemiluminescence enzyme linked immunosorbent assay for determination of tetracyclines in milk. Anal. Biochem..

[26] Wang G., Xia W.Q., Liu J.X., Wang J.P., Liu J. (2019). Directional evolution of TetR protein and development of a fluoroimmunoassay for screening of tetracyclines in egg. Microchem. J..

[27] Xia W.Q., Cui P.L., Wang J.P., Liu J. (2021). Synthesis of photoaffinity labeled activity-based protein profiling probe and production of natural TetR protein for immunoassay of tetracyclines in milk. Microchem. J..

[28] Danyi S., Degand G., Duez C., Granier B., Maghuin-Rogister G., Scippo M.L. (2007). Solubilisation and binding characteristics of a recombinant beta_2_-adrenergic receptor expressed in the membrane of Escherichia coli for the multianalyte detection of beta-agonists and antagonists residues in food-producing animals. Anal. Chim. Acta.

[29] Magnet S., Blanchard J.S. (2005). Molecular insights into aminoglycoside action and resistance. Chem. Rev..

[30] Carter A.P., Clemons W.M., Brodersen D.E., Morgan-Warren R.J., Wimberly B.T., Ramakrishnan V. (2000). Functional insights from the structure of the 30S ribosomal subunit and its interactions with antibiotics. Nature.

[31] Fosso M.Y., Li Y., Garneau-Tsodikova S. (2014). New trends in the use of aminoglycosides. Med. Chem. Commun..

[32] Sreevatsan S., Pan X., Stockbauer K.E., Williams D.L., Kreiswirth B.N., Musser J.M. (1996). Characterization of rpsL and rrs mutations in streptomycin-resistant Mycobacterium tuberculosis isolates from diverse geographic localities. Antimicrob. Agents Chemother..

[33] Kirthi N., Roy-Chaudhuri B., Kelley T., Culver G.M. (2006). A novel single amino acid change in small subunit ribosomal protein S5 has profound effects on translational fidelity. RNA.

[34] Wang G., Inaoka T., Okamoto S., Ochi K. (2009). A novel insertion mutation in streptomyces coelicolorribosomal S12 protein results in paromomycin resistance and antibiotic overproduction. Antimicrob. Agents Chemother..

[35] Suriyanarayanan B., Lakshmi P.P., Santhosh R.S., Dhevendaran K., Priya B., Krishna S. (2016). Streptomycin affinity depends on 13 amino acids forming a loop in homology modeled ribosomal S12 protein (rpsL gene) of Lysinibacillussphaericus DSLS5 associated with marine sponge (Tedaniaanhelans). J. Biomol. Struct. Dyn..

[36] Zhang H., Yang S., Ruyck K.D., Beloglazova N., Eremin S.A., DeSaeger S., Zhang S., Shen J., Wang Z. (2019). Fluorescence polarization assays for chemical contaminants in food and environmental analyses. TRAC Trends Anal. Chem..

[37] (2019). National Food Safety Standard—Maximum Residue Limits for Veterinary Drugs in Foods.

