# Supplementary material for: Development of a Fluorescence Polarization Assay for Multi-Determination of 10 Aminoglycosides in Pork Muscle Sample Based on Ribosomal Protein S12 and Studying Its Recognition Mechanism
Source: Foods. 2022 Oct 13;11(20):3196. doi: 10.3390/foods11203196 (PMC9601613; doi:10.3390/foods11203196)
Supplement: Supplementary file 1 [file foods-11-03196-s001.zip › foods-1952812-supplementary.pdf]

foods MDPI

Development of a fluorescence polarization assay for multi-determination of 10 aminoglycosides in pork muscle sample based on ribosomal protein S12 and studying its recognition mechanism

Wan Qiu Xia [1], Lei Zhang [1], Jian Ping Wang [1,2]

[1] College of Veterinary Medicine, Hebei Agricultural University, Baoding Hebei, 071000, China

[2] Veterinary Biological Technology Innovation Center of Hebei Province, Baoding Hebei 071000, China

1. MATERIALS AND METHODS

1.1 Biological materials

All the enzymes and biological reagents were molecular biology grade. IPTG (isopropyi-β-D-thirgalactopyranoside), and restriction enzymes (*Ecor I, Hind III)* were from Takara Company (Dalian, China). EasyPure Quick Gel Extraction Kit, pET-32a, Rosetta-gami(DE3), and Luria-Bertani culture medium (liquid and solid) were from TransGen Biotech (Beijing, China). SDS-PAGE gel preparation kit was from Beijing ComWin Biotech Co. Ltd (Beijing, China).

1.2 Expression procedures of *RpsL12*

The plasmids containing the express vector pET32a-*RpsL*12 were added into 500 μL of *Escherichia coli* Rosetta-gami(DE3) solution. The mixture was heat shocked for 90 seconds at 42 ºC, kept in ice water for 2 min, and cultured on Luria-Bertani (LB) liquid culture media at 37 ºC for 1 h. Then, 150 μL bacteria solution was transferred on LB solid culture media for culturation overnight (37 ºC). The single bacterial colony was transferred into 2.5 mL LB liquid culture media for culturation overnight (37 ºC, 220 rpm). When the OD_{600} value reached 0.6, 0.5 mM IPTG was added to induce protein expression (26 ºC 2 h). The bacteria solution was centrifuged at 4,000 rpm for 10 min. The supernatant was discarded, and the left sediment was resuspended in 500 μL breakage buffer (50 mM Tris, 300 mM NaCl, 0.2%Triton X-114, 0.5 mM EDTA, 2 mM DTT, pH 8.0). After the bacteria solution was sonicated for 6 min and centrifuged for 5 min (4 ºC, 12,000 rpm), the supernatant was purified with nickel-doped agarose gel FF column to obtain the *RpsL*12 protein.

1.3 Surface plasmon resonance (SPR) test

The 10 AGs diluted with DMSO were printed on the Photo-Cross-Linker Sensor CHIP™ respectively by using Biodot™ AD1520 Array Printer. At the same time, rapamycin as the positive control and DMSO as the negative control were also printed on the chip. The chip was dried under vacuum and subjected to photocrosslinking treatment under UV irradiation. Then the chip was washed with DMF, ethanol and water in turn, dried by using nitrogen stream, and inserted into the Berthold bScreen LB 991 Microarray System for SPR test. The *RpsL*12 protein was diluted with PBS to prepare the test solutions (200, 400, 800, 1600 and 3200 nM). These solutions were injected into the Berthold bScreen LB 991 Microarray System for analysis respectively. The operation conditions were: flow rate 0.5 μL/s, association time 600 s, dissociation time 360 s, temperature 4 ºC.

```
our seq.seq                   MPTINQLVRKPRQSKITKSKSPALNKGYNSFKKSLTDVKSPQKRGVCTRV 50
Bacillus subtilis.seq         MPTINQLIRKGRVSKVENSKSPALNKGYNSFKKEHTNVSSPQKRGVCTRV 50
Escherichia coli.seq          MATVNQLVRKPRARKVAKSNVPAL-------EA--C----PQKRGVCTRV 37
Enterococcus faecalis.seq     MPTINQLVRKPRKSKVEKSDSPALNKGYNSFKKTQTNVNSPQKRGVCTRV 50
Thermus thermophilus HB8.seq  MPTINQLVRKGREKVRKKSKVPAL-------KG--A----PFRRGVCTVV 37
Acinetobacter baumannii .seq  MATVNQLVRKPRARKVAKSNVPAL-------EA--C----PQKRGVCTRV 37

our seq.seq                   GTMTPRKPNSALRKYARVRLTNQIEVTAYIPGEGHNLQEHSVVLIRGGRV 100
Bacillus subtilis.seq         GTMTPKKPNSALRKYARVRLTNGIEVTAYIPGIGHNLQEHSVVLIRGGRV 100
Escherichia coli.seq          YTTTPKKPNSALRKVCRVRLTNGFEVTSYIGGEGHNLQEHSVILIRGGRV 87
Enterococcus faecalis.seq     GTMTPKKPNSALRKYARVRLSNLIEVTAYIPGIGHNLQEHSVVLLRGGRV 100
Thermus thermophilus HB8.seq  RTVTPKKPNSALRKVAKVRLTSGYEVTAYIPGEGHNLQEHSVVLIRGGRV 87
Acinetobacter baumannii .seq  YTTTPKKPNSALRKVCRVRLTNGFEVTSYIGGEGHNLQEHSVILIRGGRV 87

our seq.seq                   KDLAGVRYHIVRGALDTAGVNGRLQSRSKYGTKRPKEKK            139
Bacillus subtilis.seq         KDLPGVRYHIVRGALDTAGVENRAQGRSKYGTKKPKAK             138
Escherichia coli.seq          KDLPGVRYHTVRGALDCSGVKDRKQARSKYGVKRPKA              124
Enterococcus faecalis.seq     KDLPGVRYHIVRGALDTAGVNDRKQSRSKYGTKRPKA              137
Thermus thermophilus HB8.seq  KDLPGVRYHIVRGVYDAAGVKDRKKSRSKYGTKKPKEAAKTAAKK      132
Acinetobacter baumannii .seq  KDLPGVRYHTVRGALDCSGVKDRKQARSKYGVKRPKA              124
```

Figure S1. Amino acid sequences of the present *RpsL*12 (*Lysinibacillus sphaericus*, PDB: 7P7S) and the *RpsL*12 proteins of *Bacillus subtili* (PDB: 3J9W), *Escherichia coli* (PDB: 5JTE), *Enterococcus faecalis* (PDB: 7P7U), *Thermus thermophilus* HB8 (PDB: 2F4V), and *Acinetobacter baumannii* (PDB: 7RYF). The amino acids highlighted in dark blue are the 100% conserved residues.

Table S1. Ka, Kd, KD and KA values of the *RpsL*12 for the 10 AGs.

Drug	Ka (1/Ms)	Kd (1/s)	KD (M)	KA
Amikacin	1.69E+03	7.20E-05	4.25E-08	24.487
Apramycin	2.02E+04	2.81E-04	1.39E-08	26.102
Gentamicin	2.18E+03	1.03E-02	4.72E-06	17.693
Micronomicin	1.41E+02	5.43E-03	3.85E-05	14.666
Neomycin	3.15E+03	4.70E-05	1.49E-08	25.997
Spectinomycin	2.11E+05	1.35E-02	6.39E-08	23.900
Streptomycin	8.20E+03	3.07E-04	3.75E-08	24.670
Netilmicin	3.71E+01	1.41E-03	3.80E-05	14.684
Paromomycin	2.63E+05	1.74E-02	6.61E-08	23.851
Etimicin	5.12E+01	1.95E-02	3.81E-04	11.358

Ka, association constant.

Kd, dissociation constant.

KD = Kd/Ka, equilibrium dissociation constant.

KA=Abs (Log2(KD), absolute affinity constant.
